# Targeted sequencing of circulating cell-free DNA in stage II-III resectable oesophageal squamous cell carcinoma patients

**DOI:** 10.1186/s12885-019-6025-2

**Published:** 2019-08-20

**Authors:** Pei Meng, Jiacong Wei, Yiqun Geng, Shaobin Chen, Miente Martijn Terpstra, Qiongyi Huang, Qian Zhang, Zuoqing Su, Wanchun Yu, Min Su, Klaas Kok, Anke van den Berg, Jiang Gu

**Affiliations:** 1 Provincial Key laboratory of Infectious Diseases and Molecular Pathology, Department of Pathology and Pathophysiology, Collaborative and Creative Centre, Shantou University Medical College, Shantou, 515041 Guangdong China; 2 Department of Pathology and Medical Biology, University of Groningen, University Medical Centre Groningen, 9700RB Groningen, Netherlands; 3 Department of Genetics, University of Groningen, University Medical Centre Groningen, 9700RB Groningen, Netherlands; 4 Department of Thoracic surgery, Cancer Hospital of Shantou University, Shantou, 515041 Guangdong China; 5 Department of Pathology & Institute of Clinical Pathology, Shantou University Medical College, Shantou, 515041 Guangdong China; 6 Jinxin Research Institute for Reproductive Medicine and Genetics, Chengdu Jinjiang Hospital for Maternal and Child Health Care, 66 Jingxiu Road, Chengdu, 610066 China

**Keywords:** Oesophageal squamous cell carcinoma, Circulating cell-free DNA, Next-generation sequencing

## Abstract

**Background:**

The aim of this study was to investigate the potential of cell-free DNA (cfDNA) as a disease biomarker in oesophageal squamous cell carcinoma (ESCC) that can be used for treatment response evaluation and early detection of tumour recurrence.

**Methods:**

Matched tumour tissue, pre- and post-surgery plasma and WBCs obtained from 17 ESCC patients were sequenced using a panel of 483 cancer-related genes.

**Results:**

Somatic mutations were detected in 14 of 17 tumour tissues. Putative harmful mutations were observed in genes involved in well-known cancer-related pathways, including PI3K-Akt/mTOR signalling, Proteoglycans in cancer, FoxO signalling, Jak-STAT signalling, Chemokine signalling and Focal adhesion. Forty-six somatic mutations were found in pre-surgery cfDNA in 8 of 12 patients, with mutant allele frequencies (MAF) ranging from 0.24 to 4.91%. Three of the 8 patients with detectable circulating tumour DNA (ctDNA) had stage IIA disease, whereas the others had stage IIB-IIIB disease. Post-surgery cfDNA somatic mutations were detected in only 2 of 14 patients, with mutant allele frequencies of 0.28 and 0.36%. All other somatic mutations were undetectable in post-surgery cfDNA, even in samples collected within 3–4 h after surgery.

**Conclusion:**

Our study shows that somatic mutations can be detected in pre-surgery cfDNA in stage IIA to IIIB patients, and at a lower frequency in post-surgery cfDNA. This indicates that cfDNA could potentially be used to monitor disease load, even in low disease-stage patients.

**Electronic supplementary material:**

The online version of this article (10.1186/s12885-019-6025-2) contains supplementary material, which is available to authorized users.

## Background

Oesophageal squamous cell carcinoma (ESCC) is the most common form of oesophageal cancer and is one of the deadliest cancers worldwide [1]. Because early-stage oesophageal cancer is mostly asymptomatic, the majority of ESCC patients are diagnosed with advanced disease. Despite improvements in imaging, surgical techniques and chemoradiation therapy, effective treatment of ESCC patients remains challenging, with an overall 5-year survival of less than 30% [1]. For localized ESCC, surgery is the preferred option. However, even after radical resection, the 5-year overall survival of ESCC patients with positive lymph nodes is less than 40% [2, 3]. Moreover, the recurrence rate is also high in patients without positive lymph nodes [4]. Thus, accurate and timely detection of minimal residual disease or relapse is crucial for tailoring adjuvant therapy for a longer survival time.

Liquid biopsies have become a research hotspot for non-invasive follow-up on disease load and therapy response and for early detection of recurrence [5]. Thus far, promising results have been obtained with circulating cell-free DNA (cfDNA) [6–8]. PCR-based techniques such as droplet digital PCR (ddPCR) and PNA-mediated PCR have been used to detect recurrent mutations in *EGFR* and *KRAS* in lung cancer patients [9–12] and in *APC* in colorectal cancer [13]. Although the results are promising, these approaches require prior knowledge of the mutations present in the tumour sample. To circumvent this limitation, next generation sequencing (NGS)-based approaches using cancer hotspot panels or whole exome approaches have been applied to cfDNA. These studies have reported variable dynamic patterns in mutant allele frequencies of somatic mutations in lung cancer, breast cancer and colon cancer patients [7, 14–18]. Some studies have been carried out to monitor treatment response and disease progression by screening cfDNA samples for mutations detected in the primary tumour [5, 7]. For example, in stage II colon cancer patients, the presence of tumour DNA in cfDNA provided evidence of minimal residual disease and was associated with a higher risk of recurrence [7].

Only a few studies have focused on the analysis of cfDNA in ESCC. The amount of cfDNA was shown to be higher in ESCC patients compared to healthy controls [19]. Three to 6 months after tumour resection, the amount of cfDNA was significantly reduced, indicating that a major fraction of the cfDNA is derived from tumour cells. Another study showed the feasibility of using cfDNA before and after surgery to track tumour load [20]. Decreased mutant allele frequencies were generally observed in post-surgery plasma of 8 ESCC patients using whole exome sequencing and in 3 patients using targeted deep sequencing.

Together, these studies have indicated that circulating tumour DNA (ctDNA) can be detected in the cfDNA of ESCC patients, but additional studies are required before ctDNA can be used in routine clinical practice. In this study, we carried out targeted deep-sequencing using a cancer-related gene panel to explore the cfDNA mutation profile in stage II and III ESCC patients both pre- and post-surgery.

## Methods

### Patient selection

**Fig. 1:**
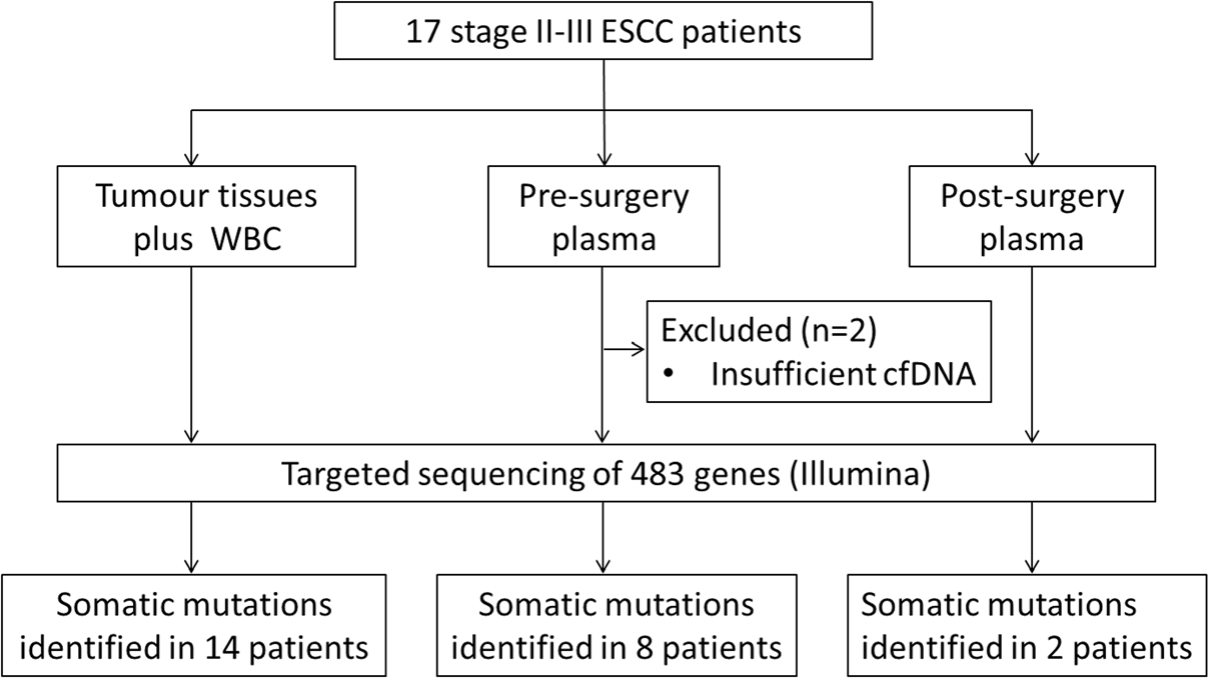
Schematic representation of the sample collection and a brief summary of the sequencing results

Seventeen ESCC patients who underwent radical tumour resection between November 1, 2013 and May 31, 2014 were included from the Shantou University cancer hospital (Fig. 1). None of the patients were treated with chemotherapy or radiotherapy before surgery. Tumour tissue samples were stored at − 80 °C and evaluated by proficient pathologists. Blood was collected 1 day before surgery and between 3 to 4 h up to 9 days after surgery. Clinical annotations were retrospectively extracted from the institutional clinical database.

### Blood sample separation

Whole blood (2-5 ml) samples were collected in EDTA tubes and processed within 2 h. After centrifugation at 900×g for 10 min, whole blood samples were separated into plasma and WBC fraction. Aliquots of plasma were subjected to two subsequent centrifugation steps at 16,000×g for 10 min at 4 °C to remove residual WBCs. Red blood cells in the WBC fraction were lysed using standard procedures. WBCs were collected by centrifugation at 600×g for 10 min and washed with PBS. All samples were stored at − 80 °C.

### DNA extraction, library preparation and sequencing

DNA isolations, library preparations and NGS of fresh frozen tumour tissues, WBCs and plasma samples were performed by Novogene (Beijing, China). In brief, the QIAamp DNA Mini Kit (Qiagen, Hilden, Germany) was used to extract DNA from fresh frozen tumour tissues. The QIAamp circulating nucleic acid kit (Qiagen) was used to isolate cfDNA from 1 to 3 ml of plasma. Genomic DNA of WBCs was extracted using the RelaxGene Blood DNA System (TianGen Biotech Co., Ltd., Beijing, China). All DNA samples were analysed using Nanodrop for purity (ratio of OD260/280) and DNA yield was quantified using the Qubit dsDNA HS assay kit on the Qubit 2.0 system (Life Technologies, Carlsbad, CA). DNA samples were stored at − 80 °C until they were subjected to NGS.

DNA input for sequencing was 500 ng for tissue samples and WBCs and about 30 ng for cfDNA samples. Exons of 483 human protein-coding genes related to cancer were included in the NGS panel designed by Novogene [21] (Additional file 1). DNA fragments were captured using Agilent SureSelect XT (Agilent, Santa Clara, CA, USA). Paired-end 150 bp reads were generated on a Hiseq2500 sequencing system (Illumina, Beijing, China).

### Data analysis

FASTQ files were obtained from the company and processed as previously described [22]. Briefly, reads were aligned to the hg19 reference genome with Burrows-Wheeler Aligner (BWA) and Genome Analysis Toolkit (GATK) [23]. Format conversion and de-duplication was performed using Picard Tools. HaplotypeCaller was used for variant calling in all samples in one workflow. The data analysis pipeline is set to report all variants with a MAF > 1%. Subsequently the pipeline reports the allele depth for these variants in all samples. Personal variants were filtered out when detected in the WBC samples at a variant allele frequency of > 0.5% in combination with a minimal variant allele count of two. In addition, we removed all variants with a sequencing depth less than 25x in WBCs, because we cannot reliably assess whether these represent personal variants or somatic mutations. Variants with a coverage of <100x or a mutant allele frequency (MAF) of < 5% in the primary tumour samples were excluded, as these variants are likely to be present in a minor subclone of the tumour. All other variants observed in the tumour samples were considered to represent somatic mutations. For all somatic mutations called in the tumour, mutations in cfDNA were determined to be present when we observed either (1) 4 altered reads with a MAF > 0.5%, or (2) > 4 altered reads and a MAF above the sequencing error background frequency, which was 0.43% per position and 0.14% per alternative nucleotide. Somatic mutations specific for cfDNA, were reported when MAF > 1% and coverage> 100. As the background rate for INDELs is much lower, we considered INDELs with two or more altered reads as true somatic mutations, irrespective of the MAF.

To further establish the reliability of the mutations reported in cfDNA in our study, we checked the read counts of the non-REF and non-variant bases by IGV. This indicates the sequencing error rate at this specific site. Prediction of pathogenicity of somatic variants was based on Combined Annotation Dependent Depletion (CADD) score [24]. We defined variants with a CADD score ≥ 20 as harmful. For the analyses of the mutational profile in tumour tissues, including the downstream pathway analysis, we focused on putative harmful mutations. DAVID v6.8 [25] was used for KEGG pathway analysis of the genes with harmful mutations. For the analyses of cfDNA, we included all somatic mutations, including non-harmful and silent mutations, as these can be equally informative for disease load.

### Statistics

For non-normally distributed data sets, median and range are given, and significance was determined by Mann-Whitney-Wilcoxon rank sum test. A *P*-value < 0.05 was considered significant.

## Results

### Patient characteristics

**Fig. 2:**
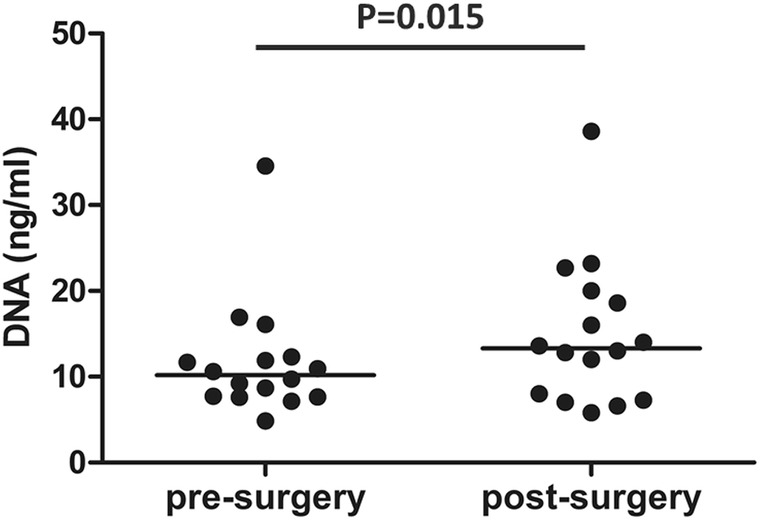
Cell-free DNA yield in pre- and post-surgery blood samples. DNA yields were calculated per millilitre of blood. Sixteen pairs of cfDNA samples were included as one of the two insufficient pre-cfDNA samples information is not available. The amount of plasma cfDNA isolated before surgery was lower than the amount obtained after surgery (p = 0.015) based on Mann-Whitney-Wilcoxon rank sum test

Characteristics of the ESCC patients are shown in Additional file 2. The cohort of 17 patients consisted of 12 males and 5 females (age range from 42 to 77 years), all diagnosed with stage II to III disease. All patients were followed-up for 24 months. Four of the 17 patients experienced disease progression at 4 to 21 months after surgery. For 2 of the 4 patients with progression, the amount of cfDNA in the pre-surgery plasma sample was too low for NGS analysis. Median cfDNA yield was 11.9 ng (range 4.86–38.6 ng) per ml of plasma. The amount of plasma cfDNA obtained before surgery was lower than after surgery (*p* = 0.015) (Fig. 2). No obvious differences in cfDNA yield were seen between samples obtained 3–4 h after surgery compared to those obtained 2–9 days after surgery.

### Overview of NGS results

A summary of the sequencing data is shown in Additional file 3. A phred quality score of 30 (Q30) was achieved for 91% of the bases. Mean target coverage of all samples was 667x and more than 95% of the target bases reached a coverage of more than 100x. The average mismatch rate per nucleotide position was 0.14% per base. Additional file 4 gives an overview of the somatic mutations detected per sample and per patient. No somatic mutations were detected in the tumour samples of three of the patients, nor in their corresponding pre- and post-surgery cfDNA samples. We therefore excluded these three patients from further analysis.

### Somatic mutations in tumour DNA

We detected a total of 131 somatic mutations with a median coverage of 348x (range 100x to 3131x) (Additional file 4). Sixty-three of the 131 (48.1%) had a CADD score > 20, indicating a putatively pathogenic effect. The median number of mutations per patient was 9 (range 2 to 17) and the median MAF was 21.0% (range 9.7 to 85.2%). *TP53* was the most commonly mutated gene, with pathogenic mutations in 14 patients, followed by mutations in *NOTCH1* (4 patients), *CDKN2A* (3 patients), *KMT2C* (2 patients) and *PTEN* (2 patients) (Table 1). Two recurrent mutations were observed one in TP53 and one in CDKN2A. Pathway analysis of the 37 genes with harmful mutations indicated a total of 30 significantly enriched pathways (*p*-value < 0.01), each encompassing 4 to 9 mutated genes (Additional file 5). These include the PI3K-Akt/mTOR (9 genes), FoxO (7 genes), Jak-STAT (7 genes), Chemokine (7 genes) and Focal adhesion (7 genes) signalling pathways and the Proteoglycans in cancer (8 genes) pathway.

**Table 1:** Overview of all recurrently mutated genes (mutated in at least 3 patients)

Gene	Patient ID	CDS mutation	Amino Acid change	CADD Score	MAF			
					tumour DNA	Pre-cfDNA	Post-cfDNA	WBC
*TP53*	ESCC01	c.1036G > T	p.Glu346*	44	24.9%	2.2%	–	–
		c.673-2A > G	.	23	43.9%	2.3%	–	–
	ESCC02	c.733G > A	p.Gly245Ser	35	43.0%	–	–	–
		c.839G > C	p.Arg280Thr	33	20.2%	–	–	–
	ESCC03	c.614A > G	p.Tyr205Cys	24	73.7%	–	–	–
	ESCC04	c.223_229delCCTGCAC	p.Pro75fs	20	76.1%	1.7%	–	–
	ESCC05	c.364_365insT	p.Thr123fs	35	85.2%	–	–	–
	ESCC06	c.551_554delATAG	p.Asp184fs	33	10.7%	–	–	–
	ESCC07	c.920-1G > T	.	25	14.4%	NA	–	–
	ESCC08	c.770_782 + 10delTGGAAGACTCCAGGTCAGGAGCC	p.Leu257fs	33	18.3%	0.9%	–	–
	ESCC09	c.643A > G	p.Ser215Gly	29	29.3%	2.3%	–	–
	ESCC10	c.481G > A	p.Ala161Thr	27	66.2%	2.8%	–	–
	ESCC11	c.844C > T	p.Arg282Trp	33	27.1%	NA	–	–
	ESCC13			33	21.9%	–	–	–
	ESCC12	c.643A > C	p.Ser215Arg	27	27.9%	–	–	–
	ESCC14	c.742C > T	p.Arg248Trp	34	27.3%	0.9%	–	–
		c.818G > A	p.Arg273His	27	15.0%	–	–	–
*NOTCH1*	ESCC01	c.4672dupG	p.Leu1559fs	35	57.6%	4.9%	–	–
		c.1070 T > C	p.Phe357Ser	29	13.6%	2.1%	–	–
	ESCC02	c.1359_1361delCAA	p.Asn454del	19	62.7%	–	–	–
	ESCC05	c.867_868insC	p.Gln290fs	29	83.2%	–	–	–
	ESCC08	c.928G > A	p.Gly310Arg	27	26.8%	0.6%	–	–
	ESCC09	c.4646G > T	p.Cys1549Phe	29	27.6%	1.8%	–	–
*KMT2D*	ESCC02	c.12823C > T	p.Gln4275*	41	43.8%	–	–	–
		c.14119C > G	p.Pro4707Ala	18	20.3%	–	–	–
	ESCC05	c.636delA	.	1	30.4%	–	–	–
	ESCC08	c.9730delG	p.Glu3244fs	35	13.5%	–	–	–
*KMT2C*	ESCC04	n.-1G > A	.	6	19.5%	–	–	–
	ESCC11	c.569G > A	p.Arg190Gln	24	15.2%	NA	–	–
	ESCC14	c.11953G > A	p.Gly3985Arg	24	12.7%	0.6%	0.4%	–
*CDKN2A*	ESCC09	c.316 + 1G > T	.	27	28.8%	2.6%	–	–
		c.172C > T	p.Arg58*	35	27.0%	1.8%	–	–
	ESCC10			35	65.5%	1.4%	–	–
	ESCC14	c.488G > A	p.Arg163Gln	34	42.6%	–	–	–
*ETV6*	ESCC01	c.329-72C > T	.	4	21.1%	–	–	–
	ESCC04	c.464-2686G > C	.	2	36.2%	0.6%	–	–
	ESCC10	c.164-14646G > A	.	2	20.3%	0.7%	–	–

* Nonsense

### Somatic mutations in pre- and post-surgery cfDNA

Part of the mutations identified in tumour samples were also identified in cfDNA. No novel mutations were found in any of the patients including the 3 patients without somatic mutations in the tumour. Five of the patients had no detectable somatic mutations in pre- and/or post-surgery cfDNA (Table 2). Seven patients had somatic mutations pre-surgery, but not in post-surgery cfDNA. One patient (ESCC14) had 4 mutations in pre-surgery cfDNA and one mutation in post-surgery cfDNA. The remaining patient (ESCC07) lacked pre-surgery cfDNA, but did have one mutation in post-surgery cfDNA. As a control for the reliability of our filtering criteria, we analysed the sequencing error read at mutant base positions of all mutations detected in the tumour samples. In all cases this was less than 4 reads in all cfDNA samples and below the MAF observed for the cfDNA sample (Additional file 6).

**Table 2:** Overview of clinical characteristics and the number of somatic mutations in tumour DNA and cfDNA

Sample ID	Stage	PFS time	Post-surgery CRT	Blood drawing time	Number of somatic mutations		
					Tumour	Pre-surgery	Post-surgery
ESCC01	PT2N0M0G2–3 IIA	5 m	N	2d	12	6	0
ESCC02	PT3N0M0G2 IIB	N	N	3-4 h	13	2	0
ESCC03	PT2N0M0G2 IIA	N	N	3-4 h	13	0	0
ESCC04	PT3N0M0G2 IIA	N	N	3-4 h	17	6	0
ESCC05	PT3N0M0G1 IIA	N	N	9d	9	1	0
ESCC06	PT3N0M0G2 IIA	N	N	5d	3	0	0
ESCC07	PT3N0M0G1 IIA	12 m	Y	3-4 h	3	NA	1
ESCC08	PT3N1M0G1 IIIB	N	N	3-4 h	9	3	0
ESCC09	PT4aN0M0G2 IIIB	N	N	3-4 h	10	8	0
ESCC10	PT4aN0M0G2 IIIB	N	N	9d	20	16	0
ESCC11	PT3N1M0G3 IIIB	4 m	N	3-4 h	5	NA	0
ESCC12	PT3N1M0G2 IIIB	N	Y	3-4 h	2	0	0
ESCC13	PT3N1M0G2 IIIB	N	Y	3-4 h	2	0	0
ESCC14	PT2N1M0G2 IIIA	N	Y	6d	13	4	1

*PFS* Progression-free survival, *N* no progression during follow-up, *m* months, *CRT* Chemoradiotherapy, *NA* Not Available

**Fig. 3:**
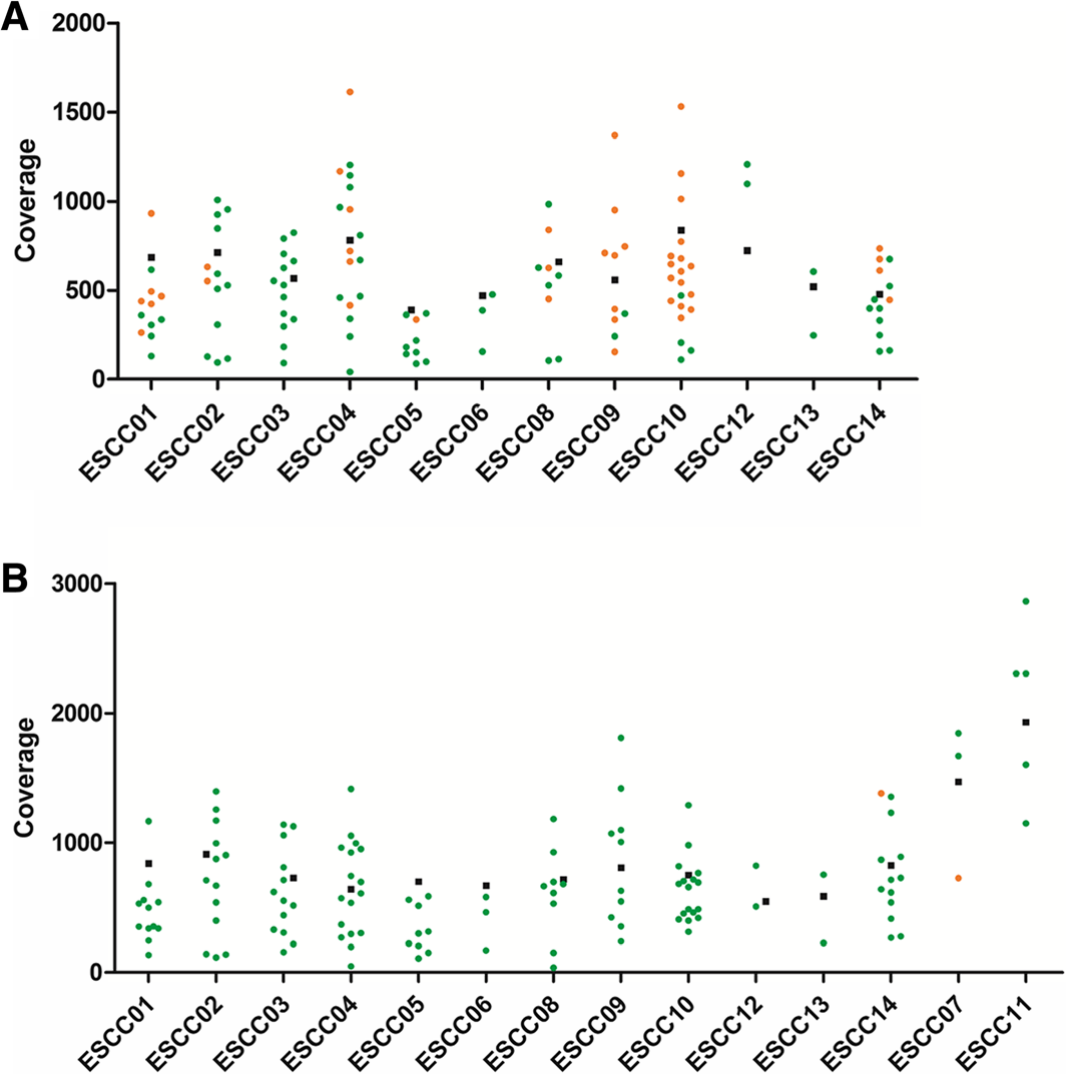
Coverage at the target regions in cfDNA samples. **a**) pre-surgery cfDNA samples. **b**) post-surgery cfDNA samples. Mean target region coverage (black squares) and the coverage for the nucleotide positions for which somatic mutations were detected in the corresponding tumour samples is indicated. Dot colours indicate coverage at the nucleotide position for which the mutant allele was (orange dots) or was not (green dots) detected in cfDNA

**Fig. 4:**
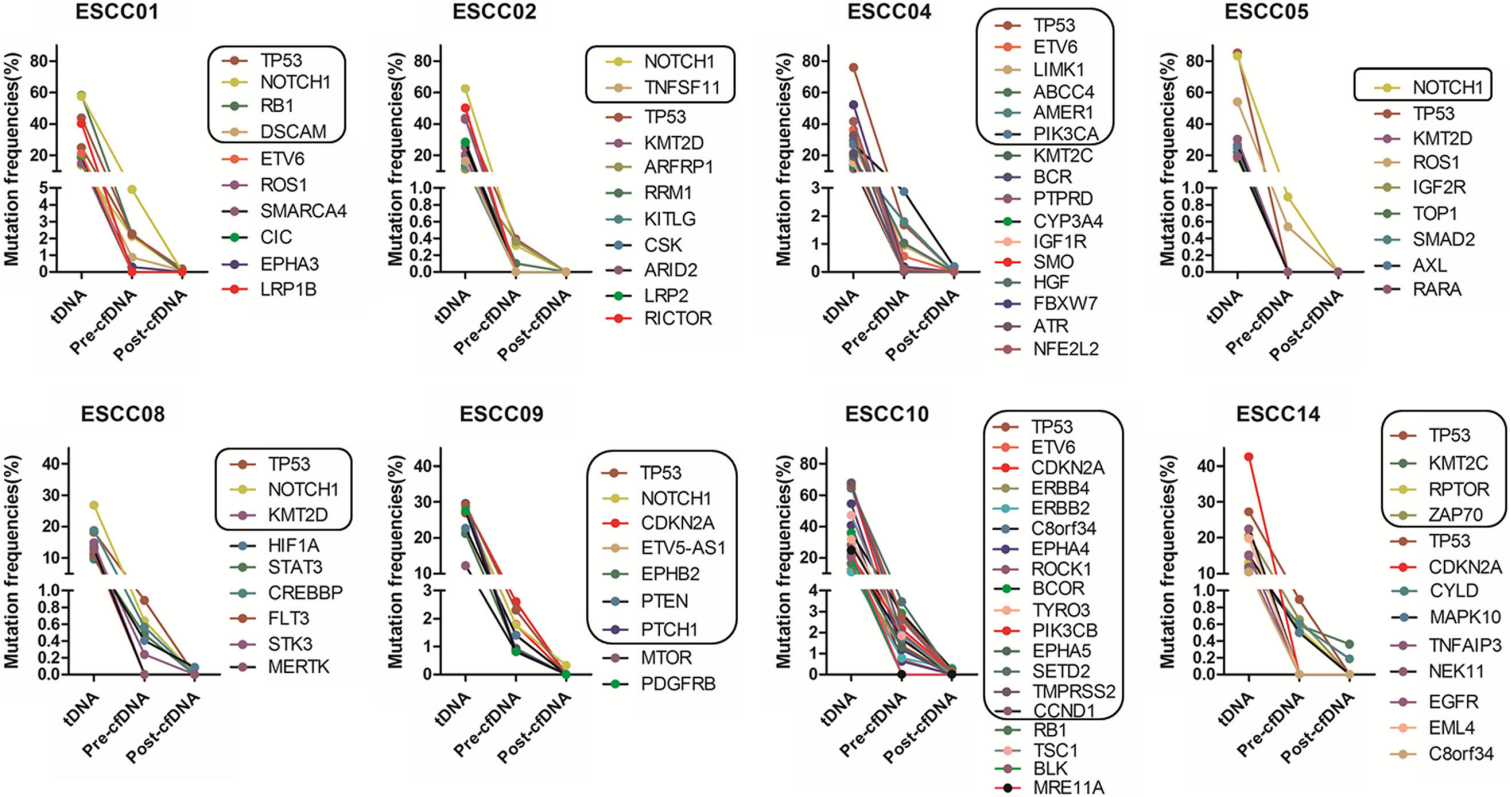
Overview of mutant allele frequencies in tumour DNA (tDNA) and cfDNA per patient. All ctDNA allele frequencies are shown, including those with altered read number and allele frequencies below our threshold. Two different mutations were detected in CDKN2A in ESCC09 and in EPHA4 in ESCC10. For all mutations, a much lower MAF was observed in post-surgery cfDNA. The black boxes indicate the tumour-specific somatic mutations that were detected in either pre- or post-surgery cfDNA plasma samples

The median number of mutations observed in pre-surgery cfDNA was 5 per patient. For two patients, a high proportion of the somatic mutations detected in the tumour were also detected in pre-surgery cfDNA (80% for both ESCC09 and ESCC10). The median on-target coverage for the cfDNA samples was 613x (range 391x to 839x) pre-surgery and 752x (range 546x to 1932x) post-surgery. There was no difference in the coverage at positions for which mutant reads were detected (median 673x, range 391x to 839x) as compared to positions for which no mutant reads were detected (median 543x, range 471x to 725x) in cfDNA (Fig. 3). The median mutant allele frequency was 1.3% in pre-surgery cfDNA (range 0.24 to 4.91%). For all somatic mutations, the MAFs in pre-surgery cfDNA were much lower than those observed in the tumour tissue (Fig. 4).

In 2 of the 14 post-surgery cfDNA samples, we identified one of the somatic mutations observed in the corresponding tumour tissue. In the remaining 12 post-surgery cfDNA samples, no mutations were observed. Mutant allele frequencies of the two mutations were 0.28% (ESCC07) and 0.36% (ESCC14). For one patient the time between surgery and blood collection was 3–4 h and for the second patient this was 6 days.

### Correlation of cfDNA mutations with clinical characteristics

We detected cfDNA mutations in pre-surgery samples from 4 out of 6 stage II and 4 out of 6 stage III patients. Only one of the three patients with disease recurrence within 1 year had pre-surgery cfDNA. In this patient, 6 of the 12 somatic mutations observed in the corresponding tumour DNA were detectable in cfDNA. For the other two patients, we only had post-surgery cfDNA, and in one of the two we detected a single mutation out of three somatic mutations detected in the tumour samples.

## Discussion

Early detection of tumour recurrence and a tool to evaluate treatment response in ESCC would allow us to optimize treatment strategy for individual patients. In the absence of effective prognostic biomarkers in ESCC [26], analysis of cfDNA might provide an easily accessible source of information to monitor disease load after surgery. In this study we performed targeted sequencing of pre- and post-surgery cfDNA and of matched tumour tissues and WBCs. Our main finding is that we were able to detect a subset of the tumour-specific somatic mutations in pre-surgery cfDNA for most of our stage II and stage III ESCC patients using as little as 3 ml blood. Most of these mutations were not detected in cfDNA of blood samples obtained as early as 3–4 h after surgery.

Even though our study cohort size is limited, this is the most extensive study thus far to compare pre- and post-surgery cfDNA in stage II and III ESCC patients. For 3 of the patients no somatic mutations were identified, despite the use of a broad cancer gene panel consisting of 483 genes. Thus, future studies should focus on a larger or ESCC-specific panel to allow detection of mutations in all patients.

Regretfully, due to lack of material we could not do an independent validation of the mutations detected in cfDNA. To overcome this shortcoming, we monitored sequencing error rates for all positions for which we identified mutations in cfDNA using our predefined criteria. This indicated that the sequencing errors did not pass our criteria. Moreover, in our previous NGS-based studies we validated close to 100% of the mutations called in the NGS data by the same pipeline for all variants with 4 or more reads by an independent technique [27, 28]. Finally, our pipeline for variant calling is based on the GATK workflow, and this pipeline was previously shown to have a sensitivity of 95% and positive predictive value of 99% [29]. Taken together, we consider the mutations in cfDNA as called in our study as reliable.

Previous studies have demonstrated that ctDNA can be detected in most advanced-stage cancer patients with high sensitivity. This allows monitoring of therapeutic response, identification of tumour-specific variants relevant for choice of therapy, and detection of acquired resistance-induced mutations. Using ddPCR, somatic mutations have been detected in cfDNA of more than 75% of patients with advanced pancreatic, ovarian, colorectal, bladder, gastroesophageal, breast, melanoma, hepatocellular and head and neck cancers, but in less than 50% of primary brain, renal, prostate or thyroid cancers [18]. Thus, it is clear that the presence of ctDNA varies between cancer types. In our study cohort, somatic mutations were found in pre-surgery cfDNA in 8 out of 12 patients. Our data support the potential of using cfDNA as a biomarker of disease load for this patient group for whom no effective biomarker is currently available. The detection rate could be higher with optimized approaches for detection of variants with low MAF and using an extended ESCC-specific gene panel.

A high-throughput sequencing approach allows detection of somatic mutations in cfDNA in a more comprehensive target region, as compared to mutation-specific PCR approaches, albeit with a somewhat lower sensitivity. With the detection of ctDNA in 4 out of 6 stage II patients, our results are comparable to the findings in previous studies focusing on stage I or II colorectal, breast, lung, ovarian and pancreatic cancer, with 43 to 71% of patients harbouring somatic mutations in cfDNA [17, 30]. This indicated the presence of ctDNA even under a low tumour burden. In colorectal cancer a higher MAF in pre-operative cfDNA has been associated with disease recurrence and overall survival [17]. In our study, a stage IIA patient with a mean MAF of 2.4% in pre-surgery cfDNA, but without any detectable mutations in post-surgery cfDNA, developed a recurrence 5 months after surgery. Additional studies are needed to further prove the potential clinical relevance of high MAF in cfDNA.

Postoperative adjuvant chemoradiotherapy was recommended to eliminate micrometastatic disease and minimal residual disease. Unfortunately, there is no effective tool to assess minimal residual disease for early tailoring of adjuvant therapy to avoid both under- and overtreatment. In addition, there is also no effective tool for early relapse surveillance prior to imaging. Detection of ctDNA after resection can point to minimal residual disease or even predict clinical relapse and poor outcome in different cancer types [7, 30, 31]. Due to the limited number of patients in our study and post-surgery treatment in some of them, we cannot reliably assess the potential clinical value of the presence of ctDNA in pre- and post-surgery cfDNA. Nevertheless, we did observe timely changes in MAF in cfDNA as early as 3 to 4 h after surgery, which is consistent with the reported half-life of cfDNA ranging from 16 min to 2 h [32, 33]. The relatively short half-life of cfDNA makes it a good biomarker to monitor dynamic changes in disease load. Cellular damage due to the surgery may lead to an increased amount of cfDNA, as we observed in some post-surgery cases. This will lead to a fractional decrease in the amount of ctDNA. Thus, both cellular damage and the short half-life of cfDNA may cause drop of the MAF to below the detection limit. So for residual disease monitoring is advisable to draw blood a few days after surgery.

Theoretically, ctDNA could be used broadly to guide treatment and to monitor for treatment resistance or cancer recurrence. CtDNA is also more specific to tumour load compared to serum-based protein biomarkers such as cancer antigen 125 in ovarian cancer patients [16], and can be used for tumours for which no serum-based protein biomarkers are available, as is the case for ESCC [34].

## Conclusion

We detected tumour-specific mutations in pre-surgery cfDNA of both stage II and stage III ESCC patients. In samples taken shortly after surgery, mutations were either undetectable or had a significantly lower MAF, which indicates that the presence of mutations in cfDNA correlates with tumour load. This implies that cfDNA may be used as a marker for the presence of tumour cells in ESCC patients. Larger studies are needed to establish the clinical applicability of cfDNA and the predictive value of treatment outcome as we only had three samples of patients that relapsed after surgery.

## Additional files

Additional file 1: Overview of the 483 genes present in the Illumina cancer gene panel. (XLSX 14 kb)

Additional file 2: Clinical and pathological features of ESCC patients. (XLSX 10 kb)

Additional file 3: Overview of the sequencing results. In this table, paired read number, Q30, aligned read number, percentage of duplicate reads, aligned unique read number and mean target coverage are listed per sample. (XLSX 18 kb)

Additional file 4: Details of all somatic mutations. Coding sequence mutations, amino acid changes, CADD score, ALT read counts and MAF for tumour, pre-surgery plasma, post-surgery plasma, and WBC are listed. (XLSX 42 kb)

Additional file 5: Overview of the pathway analysis by DAVID. All genes with predictive pathogenic mutation were analysed through DAVID. The table shows the KEGG pathways, genes in each pathway and *p*-values. (XLSX 14 kb)

Additional file 6: Background sequencing errors. Background mutant reads for each of 37 somatic SNVs (X-axes) are indicated by their absolute read number (panel A) and by their allele frequency (Panel B). Background variants are defined as the non-reference-specific and non-tumour-specific nucleotide at the position of the SNVs. The nucleotide variant with the highest read count is shown (in red) to illustrate the mismatch error rate at the positions mutated in primary tumour samples. All background variants (shown in red) were filtered out by our custom filter criteria and all variants listed as true cfDNA mutations had read counts well above these background levels (blue squares). (PDF 1561 kb)

## Data Availability

All data generated or analysed during this study are included in its supplementary information files and are available from the corresponding author on reasonable request.

## Abbreviations

cfDNA: cell free DNA
ctDNA: circulating tumour DNA
ddPCR: droplet digital PCR
EC: Oesophageal cancer
ESCC: Oesophageal squamous cell carcinoma
MAF: Mutant allele frequency
NGS: Next generation sequencing

## References

[1] Pennathur A, Gibson MK, Jobe BA, Luketich JD (2013). Oesophageal carcinoma. Lancet.

[2] Li L, Zhao L, Lin B, Su H, Su M, Xie D, Jin X, Xie C (2017). Adjuvant therapeutic modalities following three-field lymph node dissection for stage II/III esophageal squamous cell carcinoma. J Cancer.

[3] Xiao ZF, Yang ZY, Liang J, Miao YJ, Wang M, Yin WB, Gu XZ, Zhang DC, Zhang RG, Wang LJ (2003). Value of radiotherapy after radical surgery for esophageal carcinoma: a report of 495 patients. Ann Thorac Surg.

[4] Shen WB, Gao HM, Zhu SC, Li YM, Li SG, Xu JR (2017). Analysis of the causes of failure after radical surgery in patients with PT3N0M0 thoracic esophageal squamous cell carcinoma and consideration of postoperative radiotherapy. World J Surg Oncol.

[5] Heitzer E, Ulz P, Geigl JB (2015). Circulating tumor DNA as a liquid biopsy for cancer. Clin Chem.

[6] Siravegna G, Mussolin B, Buscarino M, Corti G, Cassingena A, Crisafulli G, Ponzetti A, Cremolini C, Amatu A, Lauricella C (2015). Clonal evolution and resistance to EGFR blockade in the blood of colorectal cancer patients. Nat Med.

[7] Tie J, Wang Y, Tomasetti C, Li L, Springer S, Kinde I, Silliman N, Tacey M, Wong H-L, Christie M (2016). Circulating tumor DNA analysis detects minimal residual disease and predicts recurrence in patients with stage II colon cancer. Sci Transl Med.

[8] Newman AM, Bratman SV, Wynne JF, Eclov NC, Modlin LA, Liu CL, Neal JW, Wakelee HA, Merritt RE, To J (2014). An ultrasensitive method for quantitating circulating tumor DNA with broad patient coverage. Nat Med.

[9] Thierry AR, Mouliere F, El Messaoudi S, Mollevi C, Lopez-Crapez E, Rolet F, Gillet B, Gongora C, Dechelotte P, Robert B (2014). Clinical validation of the detection of KRAS and BRAF mutations from circulating tumor DNA. Nat Med.

[10] Douillard J-Y, Ostoros G, Cobo M, Ciuleanu T, Cole R, McWalter G, Jill Walker P, Dearden S, Webster A, Milenkova T (2014). Gefitinib treatment in EGFR mutated caucasian NSCLC: circulating-free tumor DNA as a surrogate for determination of EGFR status. J Thorac Oncol.

[11] Hye-Ryoun Kim SYL, Hyun D-S, Lee MK, Lee H-K, Choi C-M, Yang S-H, Kim Y-C, Lee YC, Kim SY, Jang SH, Lee JC, Lee KY (2014). Detection of EGFR mutations in circulating free DNA by PNA-mediated PCR clamping. J Exp Clin Cancer Res.

[12] Xu J-M, Liu X-J, Ge F-J, Lin L, Wang Y, Sharma MR, Liu Z-Y, Tommasi S, Paradiso A (2014). KRAS mutations in tumor tissue and plasma by different assays predict survival of patients with metastatic colorectal cancer. J Exp Clin Cancer Res.

[13] Diehl FLM, Dressman D, He Y, Shen D, Szabo S, Diaz LA, Goodman SN, David KA, Juhl H, Kinzler KW, Vogelstein B (2005). Detection and quantification of mutations in the plasma of patients with colorectal tumors. Proc Natl Acad Sci U S A.

[14] Rothe F, Laes JF, Lambrechts D, Smeets D, Vincent D, Maetens M, Fumagalli D, Michiels S, Drisis S, Moerman C (2014). Plasma circulating tumor DNA as an alternative to metastatic biopsies for mutational analysis in breast cancer. Ann Oncol.

[15] Xu S, Lou F, Wu Y, Sun DQ, Zhang JB, Chen W, Ye H, Liu JH, Wei S, Zhao MY (2016). Circulating tumor DNA identified by targeted sequencing in advanced-stage non-small cell lung cancer patients. Cancer Lett.

[16] Forshew T, Murtaza M, Parkinson C, Gale D, Tsui DW, Kaper F, Dawson SJ, Piskorz AM, Jimenez-Linan M, Bentley D (2012). Noninvasive identification and monitoring of cancer mutations by targeted deep sequencing of plasma DNA. Sci Transl Med.

[17] SM PJ, Adleff V, Leal A, Hruban C, White J, Anagnostou V, Fiksel J, Cristiano S, Papp E, Speir S, Reinert T, Orntoft MW, Woodward BD, Murphy D, Parpart-Li S, Riley D, Nesselbush M, Sengamalay N, Georgiadis A, Li QK, Madsen MR, Mortensen FV, Huiskens J, Punt C, van Grieken N, Fijneman R, Meijer G, Husain H, Scharpf RB, Diaz LA Jr, Jones S, Angiuoli S, Ørntoft T, Nielsen HJ, Andersen CL, Velculescu VE. Direct detection of early-stage cancers using circulating tumor DNA. Sci Transl Med. 2017;9(403):eaan2415. 10.1126/scitranslmed.aan2415 PMC6714979 28814544

[18] Bettegowda C, Sausen M, Leary RJ, Kinde I, Wang Y, Agrawal N, Bartlett BR, Wang H, Luber B, Alani RM (2014). Detection of circulating tumor DNA in early- and late-stage human malignancies. Sci Transl Med.

[19] Banki FMR, Oh D, Hagen JA, DeMeester SR, Lipham JC, Tanaka K, Danenberg KD, Yacoub WN, Danenberg PV, DeMeester TR (2007). Plasma DNA as a molecular marker for completeness of resection and recurrent disease in patients with esophageal Cancer. Arch Surg.

[20] Luo H, Li H, Hu Z, Wu H, Liu C, Li Y, Zhang X, Lin P, Hou Q, Ding G (2016). Noninvasive diagnosis and monitoring of mutations by deep sequencing of circulating tumor DNA in esophageal squamous cell carcinoma. Biochem Biophys Res Commun.

[21] Yu JY, Yu SF, Wang SH, Bai H, Zhao J, An TT, Duan JC, Wang J (2016). Clinical outcomes of EGFR-TKI treatment and genetic heterogeneity in lung adenocarcinoma patients with EGFR mutations on exons 19 and 21. Chin J Cancer.

[22] Wei J, van der Wekken AJ, Saber A, Terpstra MM, Schuuring E, Timens W, Hiltermann TJN, Groen HJM, van den Berg A, Kok K. Mutations in EMT-Related Genes in ALK Positive Crizotinib Resistant Non-Small Cell Lung Cancers. Cancers (Basel). 2018;10(1):10. 10.3390/cancers10010010 PMC5789360 29300322

[23] McKenna A, Hanna M, Banks E, Sivachenko A, Cibulskis K, Kernytsky A, Garimella K, Altshuler D, Gabriel S, Daly M (2010). The genome analysis toolkit: a MapReduce framework for analyzing next-generation DNA sequencing data. Genome Res.

[24] Kircher M, Witten DM, Jain P, O'Roak BJ, Cooper GM, Shendure J (2014). A general framework for estimating the relative pathogenicity of human genetic variants. Nat Genet.

[25] Huang da W, Sherman BT, Lempicki RA (2009). Systematic and integrative analysis of large gene lists using DAVID bioinformatics resources. Nat Protoc.

[26] Qing T, Zhu S, Suo C, Zhang L, Zheng Y, Shi L (2017). Somatic mutations in ZFHX4 gene are associated with poor overall survival of Chinese esophageal squamous cell carcinoma patients. Sci Rep.

[27] Saber A, Hiltermann TJN, Kok K, Terpstra MM, de Lange K, Timens W, Groen HJM, van den Berg A (2017). Mutation patterns in small cell and non-small cell lung cancer patients suggest a different level of heterogeneity between primary and metastatic tumors. Carcinogenesis.

[28] Liu Y, Abdul Razak FR, Terpstra M, Chan FC, Saber A, Nijland M, van Imhoff G, Visser L, Gascoyne R, Steidl C (2014). The mutational landscape of Hodgkin lymphoma cell lines determined by whole-exome sequencing. Leukemia.

[29] McCormick RF, Truong SK, Mullet JE (2015). RIG: Recalibration and interrelation of genomic sequence data with the GATK. G3 (Bethesda, Md).

[30] Sausen M, Phallen J, Adleff V, Jones S, Leary RJ, Barrett MT, Anagnostou V, Parpart-Li S, Murphy D, Kay Li Q (2015). Clinical implications of genomic alterations in the tumour and circulation of pancreatic cancer patients. Nat Commun.

[31] Majure M, Logan AC (2016). What the blood knows: interrogating circulating tumor DNA to predict progression of minimal residual disease in early breast cancer. Ann Transl Med.

[32] Rostami A, Bratman SV (2017). Utilizing circulating tumour DNA in radiation oncology. Radiother Oncol.

[33] Diehl F, Schmidt K, Choti MA, Romans K, Goodman S, Li M, Thornton K, Agrawal N, Sokoll L, Szabo SA (2008). Circulating mutant DNA to assess tumor dynamics. Nat Med.

[34] Qi YJ, Chao WX, Chiu JF (2012). An overview of esophageal squamous cell carcinoma proteomics. J Proteome.

