# Supplementary figures and images for: Targeted sequencing of circulating cell-free DNA in stage II-III resectable oesophageal squamous cell carcinoma patients
Source: BMC Cancer. 2019 Aug 20;19:818. doi: 10.1186/s12885-019-6025-2 (PMC6701116; doi:10.1186/s12885-019-6025-2)

A

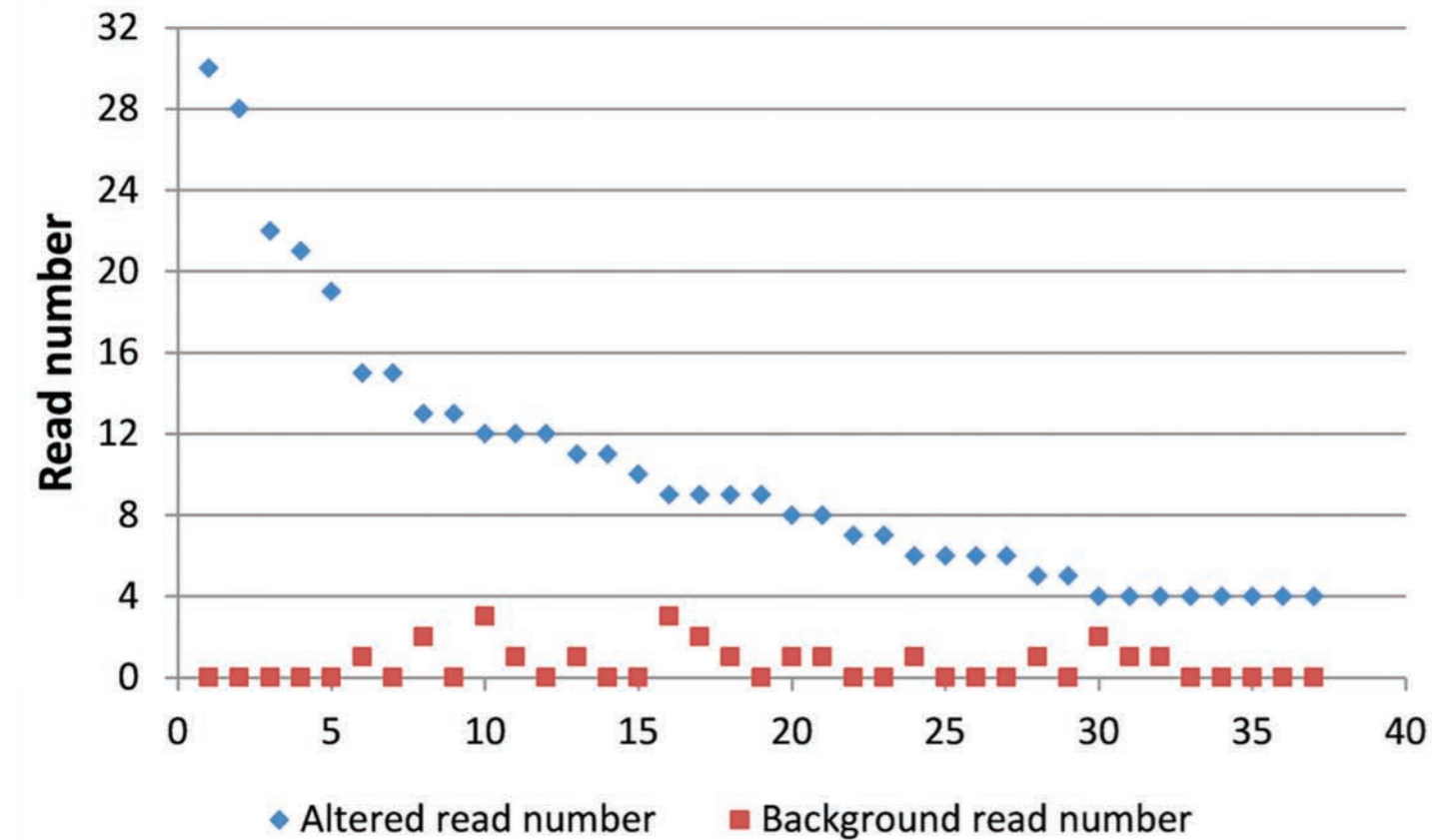

B

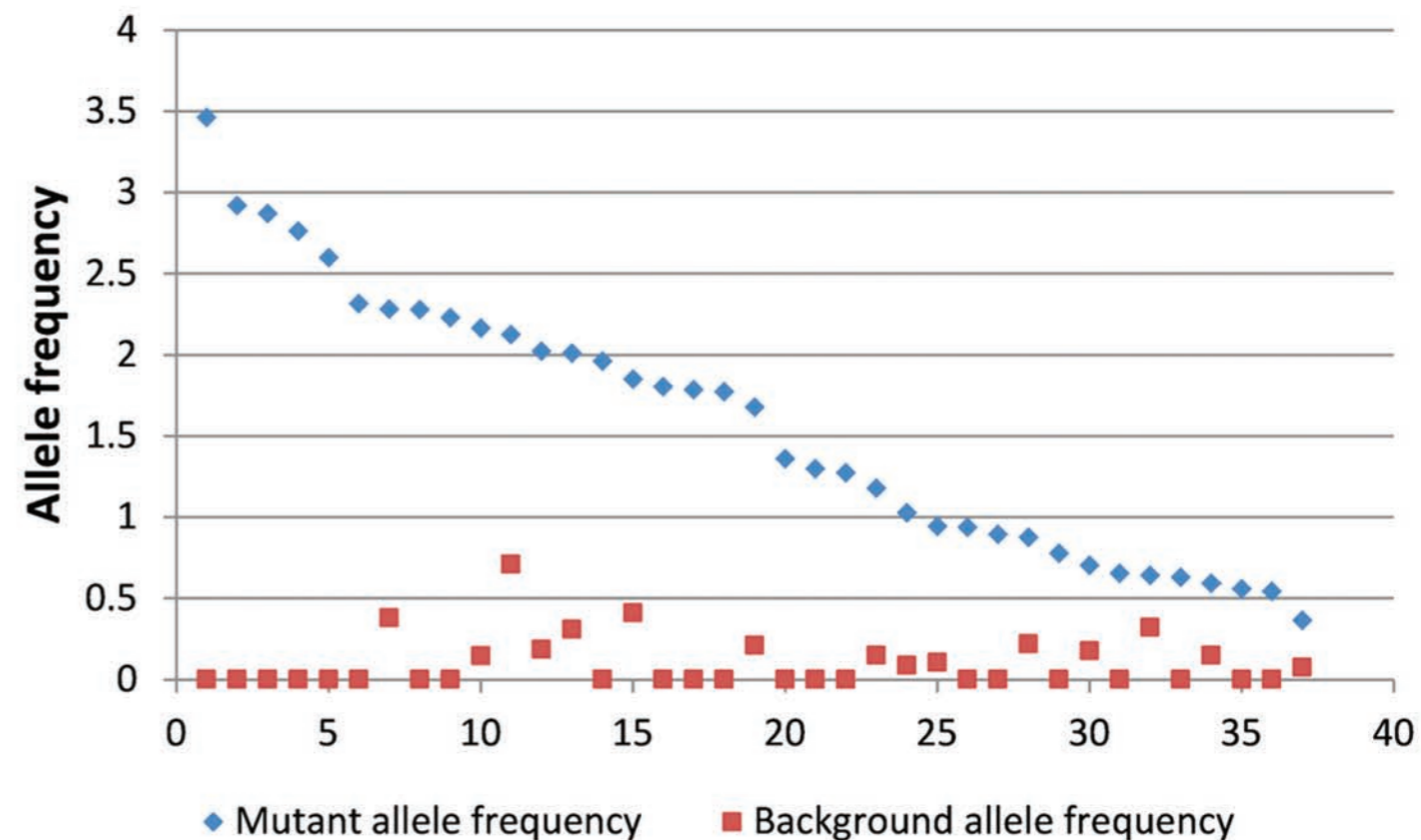

Supplement: Supplementary file 6 — Background sequencing errors. Background mutant reads for each of 37 somatic SNVs (X-axes) are indicated by their absolute read number (panel A) and by their allele frequency (Panel B). Background variants are defined as the non-reference-specific and non-tumour-specific nucleotide at the position of the SNVs. The nucleotide variant with the highest read count is shown (in red) to illustrate the mismatch error rate at the positions mutated in primary tumour samples. All background variants (shown in red) were filtered out by our custom filter criteria and all variants listed as true cfDNA mutations had read counts well above these background levels (blue squares). (PDF 1561 kb) [file 12885_2019_6025_MOESM6_ESM.pdf]
